# Bi-nasal sectors of ganglion cells complex and visual evoked potential amplitudes as biomarkers in pituitary macroadenoma management

**DOI:** 10.3389/fnint.2022.1034705

**Published:** 2022-11-24

**Authors:** Odelaisys Hernández-Echevarría, Elizabeth Bárbara Cuétara-Lugo, Mario Jesús Pérez-Benítez, Julio César González-Gómez, Héctor Raúl González-Diez, Carlos E. Mendoza-Santiesteban

**Affiliations:** ^1^Department of Neuro-ophthalmology, Cuban Institute of Ophthalmology “Ramón Pando Ferrer”, University of Medical Sciences of Havana, Havana, Cuba; ^2^Department of Research and Academic, National Institute of Oncology and Radiobiology, University of Medical Sciences of Havana, Havana, Cuba; ^3^Department of Science and Technology, University of Informatics Sciences, Havana, Cuba; ^4^Department of Neuro-ophthalmology, Bascom Palmer Eye Institute, University of Miami, Miller School of Medicine, Coral Gables, FL, United States

**Keywords:** pituitary macroadenoma, biomarkers, visual evoked potential, ganglion cell complex, diagnosis, follow-up

## Abstract

The study aimed to evaluate the retinal ganglion cell structure using optical coherence tomography and the visual pathway function employing visual evoked potentials in the diagnosis and monitoring of patients with pituitary macroadenoma. A descriptive, cross-sectional, and longitudinal study (3 and 12 months follow-up) was conducted on forty-two patients. Thirty-five age-matched healthy controls were used in the cross-sectional one. Full neuro-ophthalmological evaluation (structural and functional) was carried out including global and segmented retinal nerve fiber layer/ganglion cell complex analysis and amplitude and latency of P100 component in the electrophysiology. Statistical data analysis was conducted with R version 3.6.3 and Python version 3.8. Associations were evaluated using Spearman’s correlations. Amplitude sensitivities were 0.999, and bi-nasal sectors of ganglion cell complex thickness specificities were 0.999. This structural parameter had the highest diagnostic value (area under curve = 0.923). Significant associations were found between bi-nasal sectors with amplitude at 12′ (rho > 0.7, *p* < 0.01) and median deviation of the visual field (rho > 0.5, *p* < 0.01) at 3 months. Pre-surgical values of bi-nasal sectors and amplitude can predict post-surgically median deviation and amplitude (Oz, 12′) at 3 months with *r*^2^ > 0.5. Bi-nasal sectors of ganglion cell complex and visual evoked potentials P100 amplitude are efficient biomarkers of visual pathway damage for pituitary macroadenoma patients’ management. Pre-surgical values of the bi-nasal sector and visual evoked potentials’ amplitude could help to predict the restoration of parvocellular pathway traffic after decompression.

## Introduction

Pituitary adenomas are the third most common type of brain tumor, accounting for 10–15% of all brain tumors. They are the most frequent type of neuroendocrine tumor (1/2688-1/865) originating in the central nervous system. Half of them are considered pituitary macroadenoma (PMA) (>10 mm in diameter) (Molitch, 2017). In Cuba, the annual incidence of PMA and the mortality rate is unknown. Slow and progressive vision loss is the main reason for a consultation with neuro-ophthalmology services. Magnetic resonance imaging (MRI) is the gold-standard diagnostic test for PMA due to its high resolution, soft tissue contrast, multiplanar capability, and the absence of ionizing radiation (Chaudhary and Bano, 2011; Gadelha et al., 2021; Pihl-Jensen et al., 2021). MRI identifies the presence of PMA but does not provide precise information about the magnitude of chiasm and optic nerve compression, nor of its repercussion on visual function (Castro Revollo and Contreras Molina, 2012).

The main neuro-ophthalmologic finding in PMA is the visual field defect (50–96%), which results classically in bitemporal hemianopia. This type of defect is not always congruent, and other scotomas may appear depending on the anatomy of the chiasm and sellar region (Racette et al., 2016). Conversely, up to 30% of patients with PMA show no visual field abnormalities due to the size, location, and tumor growth direction. Visual field defects are generally detected when at least 30–50% of the retinal ganglion cells are damaged (Lachowicz and Lubiński, 2018). Visual field (VF) is a subjective test, and its results depend on patients’ cooperation.

In recent years, the parameters determined by optical coherence tomography (OCT) and visual evoked potential (VEP) have been used as biomarkers for the diagnosis and progression monitoring of neurodegenerative diseases (Mendoza-Santiesteban et al., 2014, 2015). OCT evaluates the retinal structure, whereas VEP assesses the visual pathway function, in an objective manner. A few publications had explored structural and functional associations (Costello and Burton, 2018; Kim et al., 2018; Heidari et al., 2019).

The evaluation of visual function using VEP offers an alternative for the characterization and personalized management of patients with PMA (Taghvaei et al., 2019; Popescu et al., 2021). VEPs objectively assess the function of the optic nerve and chiasm and eliminate patient-dependent subjectivity and cooperation factors that affect automatic perimetry (Taghvaei et al., 2019). In clinical practice, the positive wave P100 is frequently analyzed because it is constant and easy to identify (Popescu et al., 2021). According to Lachowicz and Lubiński, pattern VEPs are objective, reproducible, and more sensitive than other classical VEPs or psychophysical tests of visual function (Lachowicz and Lubiński, 2018). Prolonged latency indicates nerve impulse conduction disorder and a decrease in amplitude indicates axonal loss due to optic nerve compression or ischemia (Echevarría et al., 2015; Nowicki et al., 2020). In PMA, as a result of visual pathway compression, VEP amplitudes can be affected by nerve conduction blockade of the damaged fibers. Latency is also delayed due to slow conduction through demyelinated segments of the nerve fibers. In patients with PMA, detecting early visual pathway dysfunction may modify medical or surgical treatment and reduce the incidence of irreversible optic nerve damage (Lachowicz and Lubiński, 2018). In theory, VEP could represent an extension of the clinical examination, but they are not yet routinely performed (Lachowicz and Lubiński, 2018; Taghvaei et al., 2019; Popescu et al., 2021).

On the other hand, retinal and optic nerve structures can be objectively assessed using OCT, a computer-assisted, *in vivo*, non-invasive, and non-contact technique that provides high-resolution cross-sectional images of the human retina. The evaluation of the retinal nerve fiber layer thickness (RNFL) and ganglion cell complex (GCC) by OCT has been used as a diagnostic tool for the visual function of tumors affecting the visual pathway (Banc et al., 2018; Chwalisz et al., 2018). The predictive ability of RFNL thickness for postoperative visual function has been confirmed by numerous publications (Monteiro et al., 2014; Tieger et al., 2017; Wang et al., 2020; Lee et al., 2021). Although previous studies have investigated the predictive ability of OCT parameters, no emphasis has been placed on macular parameters, particularly the segmentation of the GCC. The studies above have been limited by sample size and follow-up period.

Recent publications support its use to assess trans-synaptic degeneration in visual pathway tumors, but there has not been consensus on which is the biomarker of choice (Lithgow et al., 2019; Wang et al., 2020). In PMA, if the condition is not treated promptly, several visual and neurological complications may develop. That is why the neuro-ophthalmologic evaluation of the retinal ganglion cell structure and objective visual function may allow the stratification of patients to decide treatment options. The present study is aimed to evaluate the retinal ganglion cell structure using OCT and the visual pathway function employing VEP in patients with PMA. It will also assess the relation between such markers and predictive values for visual recovery.

## Materials and methods

### Study design

A descriptive, cross-sectional, and the longitudinal study was performed using forty-two patients diagnosed with PMA from the neuro-ophthalmology service of the Cuban Eye Institute “Ramon Pando Ferrer” (CIORPF) from March 2017 to June 2021. The research was conducted according to the principles established in the Helsinki Declaration 7th Brazil revision, 2013 (World Medical Association [WMA], 2013). The Institutional Review Bureau of CIORPF (#12/2017) and the Ethics Committee (#27/2017) approved the present research. Patients and healthy controls expressed their willingness to participate in the study by signing the informed consent.

#### Participants. Inclusion and exclusion criteria

Patients older than 18 years of age of both genders with suggestive PMA symptoms were included. Magnetic resonance imaging (MRI) confirmation and best-corrected visual acuity (BCVA) better than 0.5 logMAR in at least one eye were indispensable for conducting visual field 32 dynamic strategies in Octopus equipment and pVEP. When both eyes of a patient fulfill the criteria, they were included since inter-eye correlation analysis revealed no correlation. For the cross-sectional study, controls were healthy volunteers matched by age and gender, who express their wiliness to participate in the study.

Exclusion criteria were spherical refractive error outside the range of plus 5D or greater than 2D astigmatism, unreliable preoperative visual field testing (defined as more than 25% false positive, false negative, or loss of fixation rate), anterior ophthalmologic segment disease that prevents fundus exam, and retina or optic nerve disease and non-attendance of the patients.

This study had two parts. First, a cross-sectional evaluation of five patients with PMA was excluded from the original 42-patient sample due to the lack of appropriate age-matched controls. Thus, 57 eyes, from 37 patients with pre-surgical PMA to assess the proposed biomarkers’ diagnostic value, were evaluated. Both eyes of patients were included when fitted inclusion criteria. It is recommended to use both eyes data when there is a low degree of inter-eye correlation regardless of the impact that ignoring such correlation could have on confidence interval and p-values (Zhang and Ying, 2018). Thirty-five healthy volunteers were selected by propensity score matching as controls. Since they had normal vision, there had a strong inter-eye correlation. That is why only one randomly selected eye was included per subject. Such a decision was based on Ying et al. statements about statistical analysis for correlated binary eye data (Ying et al., 2018). When there is a high degree of inter-eye correlation, it is recommended to use one-eye data since second-eye information may not add much statistical information (Zhang and Ying, 2018). Second, a longitudinal evaluation for the analysis of visual recovery was carried out in 61 eyes from 42 patients with PMA, and some eyes were excluded due to pre-surgical BCVA which is lower than the required for electrophysiology and visual field selected protocols. Non-attendance of the patients to one of the follow-up consultations result implied a reduction in sample size in follow-up evaluations.

### Clinical evaluation

A complete neuro-ophthalmological examination was performed on all patients including detailed clinical history and examination. Exams included BCVA (Bailey-Lovie logMAR chart, Australia), color vision (CV, Ishihara 16 plates, Tokyo, Japan), contrast sensitivity (CS, Pelli-Robson, NY, USA), intraocular pressure assessment by applanation tonometry (Haag Strait, Berne, Switzerland), pupillary reflexes, confrontation visual fields, ocular motility, Hertel’s exophthalmometry, anterior segment slit-lamp bio-microscopy, and fundus examination using indirect binocular ophthalmoscopy with fully dilated pupils. Automated visual fields were performed using Octopus 101 perimeter (32 programs, Dynamic strategy, Haag Strait, Berne, Switzerland), and median deviation (MD) was the parameter of interest.

### Magnetic resonance imaging

Brain and orbit MRI with and without gadolinium intravenous contrast were performed in all patients, as described elsewhere, to confirm PMA diagnosis and the likely compression of the visual pathways. MRI sequences included axial, coronal, and sagittal slices at 3 mm and T1, T2, Flair, and Stir sequences. The same protocol was also used in the postoperative follow-up (Gadelha et al., 2021).

### Visual evoked potentials

Pattern VEP was obtained using an RETI-port/scan system (Roland Consult, Brandenburg, Germany) and recorded monocularly in all subjects following the standards of the International Society for Clinical Electrophysiology of Vision (ISCEV) (Odom et al., 2016; Hamilton et al., 2021), without mydriasis and with optimal refractive correction. A three-channel montage was used with occipital electrodes in Oz, O1, and O2, frontal reference electrode (Fz), and mid-frontal electrode as a ground (Cz). Latency to peak and the amplitude of the P100 component as well as inter-ocular differences (Odom et al., 2016; Robson et al., 2018).

### Optical coherence tomography

Optical Coherence Tomography was performed in all visits to study the retinal structure (Cirrus-5000, Carl Zeiss Meditec, California, USA). Images were acquired in a darkened room, in a seated position. Subjects were instructed to fixate their gaze on a green target during the scan. Each subject had both eyes scanned three times using two standard acquisition protocols: macular cube (512 × 128 line scans) and optic disk cube (200 × 200 line scans). The scanned area was a 6-mm cube without signal averaging. The quality of the obtained images was assessed by evaluating the signal strength (a value from 0 to 10 in arbitrary units), and only scans with signal strength above 6 units were included in the analysis. Parameters such as RNFL thickness and global GCC thickness were obtained using two automatic segmentation algorithms and expressed in micrometers (μm). The global GCC thickness was obtained as the average of the specific thicknesses at different locations around the foveal center such as temporal-superior, superior, nasal-superior, nasal inferior, inferior, temporal-inferior, and global. The RNFL was also measured globally and at four different peripapillary locations (temporal, superior, nasal, and inferior). The bi-nasal sectors of GCC (bi-nasal of GCC) were defined as the average of inferonasal and superonasal sectors.

### Postoperative evaluation

**FIGURE 1:**
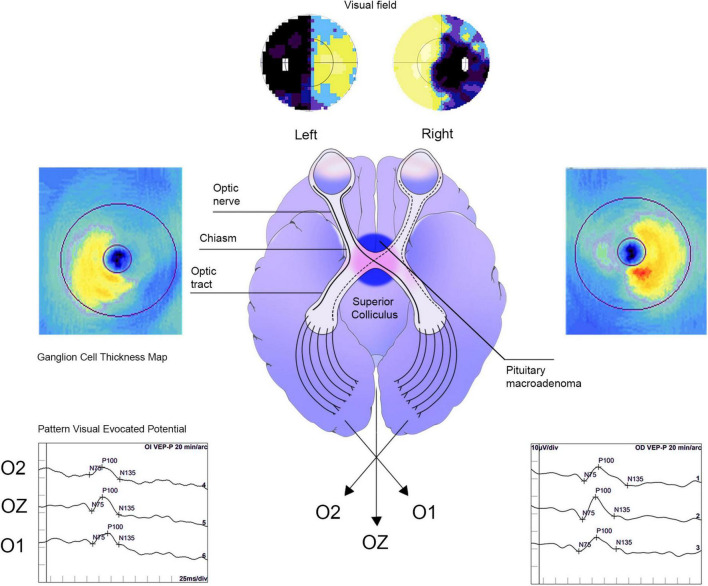
Schematic representation of neuro-ophthalmological examination of a pituitary macroadenoma patient. The pre-surgical image is related to a 61-year-old patient. Bitemporal incongruent heteronymous hemianopia was observed in VF. Thickening of the global ganglion cell complex, particularly in the bi-nasal region and vertical meridian respect in the optical coherence tomography map, was also evidenced. In addition, visual evoked potentials displayed a latency delay and reduced amplitude of the P100 wave.

Once the diagnosis of PMA was confirmed, all patients were referred to the National Minimal Access Center in Havana, Cuba, where transsphenoidal surgery for tumor removal was performed. Patients were reassessed at 3 months and 1 year after surgery for predictive purposes using the same pre-surgical protocol. Figure 1 is illustrative of the rationale of the study.

### Statistical analysis

Statistical data analysis was conducted in an R Studio working interface version 1.4.1717 with statistical program R version 3.6.3 of 2020-02-29 and Python version 3.8. For the normality distribution of data and homoscedasticity testing, “*nortest*” (Shapiro–Wilk test for up to 50 data samples and Kolmogorov–Smirnov test for larger samples) and “car” packages (Levene’s test) were used, respectively. In most of the cases, non-parametric tests were run. Inter-eye correlations were analyzed using the Wilcoxon test; when *r* ≥ 0.5, only one eye was included. Matching was carried out with propensity score matching software. Thus, the results are presented using “eye” as the unit of analysis. Spearman’s tests were performed to identify associations between parameters for predictive purposes. To integrate both eyes measurements, which means individuals as unit of analysis, generalized estimating equations (GEE) package version 1.3–2 was used. It evaluates linear relationships assuming normally distributed errors for continuous dependent measures and logistic regression with binomial errors for dichotomous dependent measures (Wang, 2014; Ying et al., 2018). Pandas, NumPy, SciPy, and statsmodels.api were run for data management and statistical analysis while matplotlib.pyplot and seaborn were used for graphic design.

## Results

### Cross-sectional study

#### Subject characteristics at baseline

To assess the diagnostic value of the proposed biomarkers, our sample included 57 eyes from 37 patients with PMA and 35 eyes from the same number of healthy controls. Age and gender distribution were 50.8 ± 11.4, 21 female/16 male patients, for patients with PMA, and 50.5 ± 11.6, 21 female/14 male patients, for controls (Table 1). Only an eye per individual from the control group was used due to the high inter-eye correlation. Since there was no correlation for patients with PMA, both eyes were included when available (57 eyes).

**TABLE 1:** Neuro-ophthalmological evaluation.

Parameter (unit)		Healthy volunteers		PMA patients		*P*-value student test	AUC	GEE coefficient	
		N (eyes)	Media ± sd	N (eyes)	Media ± sd			Left eye	Right eye
MD (dB)		28	–2.1 ± 1.6	55	10.8 ± 7.0	8.5 × 10^–12^***	0.94	2.2	1.7
BCVA (VAR/LogMAR)		27	98 ± 4/**0**	52	83 ± 19/**0.4**	3.4 × 10^–7^***	0.83	–2.5	–2.6
CV			21 ± 0	57	11 ± 8	9.6 × 10^–10^***	0.90	–5.9	–4.8
CS 1m			1.50 ± 0.30	54	1.00 ± 0.45	5.6 × 10^–8^***	0.86	–1.0	–3.4
CS 3m			1.50 ± 0.15	29	1.00 ± 0.45	1.6 × 10^–10^***	0.93	–2.2	–2.0
VEP Latency (ms)	OZ-60	16	106 ± 5	29	119 ± 16	7.7 × 10^–5^***	0.86	2.1	0.1
	OZ-20		111 ± 7		122 ± 14	1.8 × 10^–3^**	0.72	1.2	0.2
	OZ-12		120 ± 10		126 ± 14	9.6 × 10^–2^	0.65	0.3	0.6
	O1-60		107 ± 8		121 ± 18	6.3 × 10^–4^***	0.81	0.7	1.0
	O1-20		111 ± 10		123 ± 15	4.4 × 10^–3^**	0.66	0.9	–0.0
	O1-12		116 ± 13		127 ± 16	1.4 × 10^–2^*	0.72	1.1	–1.0
	O2-60		109 ± 7		120 ± 16	4.4 × 10^–3^**	0.76	1.3	–0.1
	O2-20		110 ± 8		121 ± 15	2.1 × 10^–2^*	0.71	1.1	–0.3
	O2-12		114 ± 9		122 ± 27	1.5 × 10^–2^*	0.72	1.0	–0.4
VEP Amplitude (μV)	OZ-60	16	14.5 ± 4.7	29	4.2 ± 2.4	2.8 × 10^–7^***	0.84	–1.5	–4.1
	OZ-20		15.6 ± 5.1		3.9 ± 2.8	1.4 × 10^–10^***	0.86	–1.6	–4.2
	OZ-12		15.7 ± 7.2		3.7 ± 3.1	2.2 × 10^–7^***	0.84	–3.0	–1.4
	O1-60		10.8 ± 2.9		3.5 ± 2.2	6.0 × 10^–10^***	0.85	–3.3	–0.9
	O1-20		11.8 ± 3.4		3.0 ± 2.0	6.9 × 10^–8^***	0.87	–1.8	–0.4
	O1-12		11.6 ± 4.9		4.0 ± 6.4	6.2 × 10^–7^***	0.93	–13.6	–3.7
	O2-60		10.9 ± 4.1		3.6 ± 2.1	4.4 × 10^–7^***	0.89	–6.1	–0.1
	O2-20		11.4 ± 3.7		2.8 ± 1.8	1.4 × 10^–11^***	0.87	–0.6	–0.6
	O2-12		11.0 ± 4.9		2.8 ± 2.0	1.6 × 10^–9^***	0.91	–0.5	–3.0
OCT RNFL (μm)	RFNL	35	101 ± 11	46	81 ± 15	1.3 × 10^–9^***	0.79	–1.1	–1.4
	TS		64 ± 11		51 ± 10	5.3 × 10^–7^***	0.80	–0.6	–1.9
	SS		122 ± 13		101 ± 25	1.5 × 10^–4^***	0.72	–0.9	–0.7
	NS		82 ± 12		64 ± 13	2.7 × 10^–8^***	0.83	–1.6	–0.6
	IS		134 ± 19		107 ± 24	2.1 × 10^–7^***	0.78	–0.6	–1.3
OCT GCC (μm)	Global GCC	35	85 ± 5	46	67 ± 12	1.9 × 10^–10^***	0.86	–1.5	–2.0
	RS1/LS3		85 ± 6		71 ± 12	3.7 × 10^–7^***	0.80	–0.1	–3.6
	RS2/LS2		85 ± 5		64 ± 12	2.6 × 10^–10^***	0.86	–1.9	–2.2
	RS3/LS1		86 ± 5		62 ± 13	4.8 × 10^–11^***	0.87	–2.8	–0.9
	RS4/LS6		85 ± 5		60 ± 13	1.8 × 10^–12^***	0.93	–4.4	–0.8
	RS5/LS5		84 ± 6		67 ± 12	1.3 × 10^–9^***	0.87	–0.5	–2.5
	RS6/LS4		86 ± 6		76 ± 13	7.9 × 10^–5^***	0.67	1.8	–6.2
	bi-nasal of GCC		87 ± 6		61 ± 13	4.6 × 10^–12^***	0.92	–2.0	–2.1

Values correspond to 57 eyes of 37 patients with pre-surgical pituitary macroadenoma and 35 eyes of 35 healthy volunteers selected by propensity score matching. VEP, visual evoked potential. OCT, optical coherence tomography. MD, median deviation. BCVA, best-corrected visual acuity. CV, color vision. CS 1m, contrast sensitivity at 1m. CS 3 m, contrast sensitivity at 3m. RNFL, retinal nerve fiber layer. Global GCC, global ganglion cell complex. Bi-nasal of GCC, bi-nasal sectors of GCC. O1. O2. Oz derivations. 60′-20′-12′ frequencies of stimulus. ***: *p* < 0.001, **: *p* < 0.01, *: *p* < 0.05.

#### Clinical examination

Perimetry is a subjective evaluation of visual function since it depends on patient cooperation. The most useful parameters in clinical practice are median sensitivity and MD of the visual field. MD is the average of all local defects of retinal sensitivity. This parameter is not age-related, and normal values are in the interval of 0 ± 2 dB, in 90% of normal visual fields. Generally, PMA patients exhibit bitemporal hemianopia which corresponds to pathological MD values over 3 dB. After successful chiasmal decompression surgery, the expected result is MD reduction. Descriptive statistics of perimetry and visual psychophysics are summarized in Table 1. Visual field mean deviation (MD) of PMA patients in the pre-surgical examination (10.8 ± 7.0) was higher than healthy individual values (-2.1 ± 1.6), *p* < 0.001. BCVA, CV, CS 1m, and CS 3 m were also significantly different (*p* < 0.001).

#### Visual evoked potentials

The VEP amplitudes in Oz for all spatial frequencies of the stimulus were significantly decreased in patients with PMA compared to healthy volunteers (OZ-60′: 4.2 ± 2.4 vs. 14.5 ± 5 μV; OZ-20′: 3.9 ± 2.8 vs. 16.0 ± 5.0 μV; OZ-12: 3.7 ± 3.1 vs. 16.0 ± 7.0 μV, *p* < 0.001). Latency in Oz was increased only for 60′ (*p* < 0.001) and 20′ (*p* < 0.01). Table 1 includes other significant descriptive statistics of VEP in O1 and O2.

#### Optical coherence tomography

**FIGURE 2:**
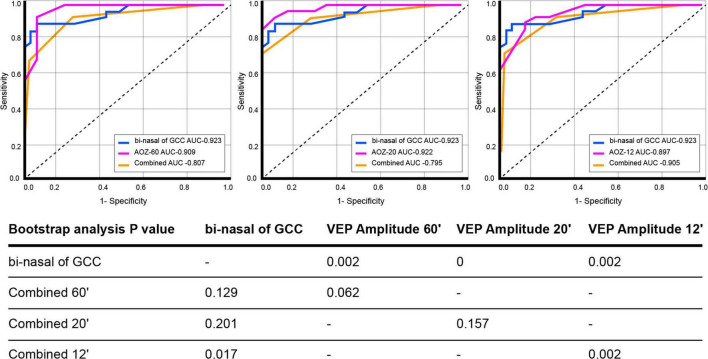
ROC curves of pre-surgical visual evoked potential amplitude (Oz 60′, 20′, and 12′), pre-surgical bi-nasal of ganglion cell complex, and combined. P related to bootstrap analysis with 10,000 iterations. Comparison between curves revealed that the bi-nasal of the ganglion cell complex is the most reliable biomarker for pituitary macroadenoma diagnosis. Visual evoked potential amplitude OZ at 60′ and 20′, when optical coherence tomography evaluation with ganglion cell complex protocol is not possible, is the diagnosis biomarker of choice.

Global GCC and bi-nasal of GCC were significantly decreased in the PMA group compared to controls (67 ± 12 vs. 85 ± 5 μm, and 61 ± 13 vs. 87 ± 6 μm, *p* < 0.001). Table 1 summarizes descriptive statistics of OCT parameters referred to RNFL and global GCC protocols. The diagnostic value of the proposed biomarkers is presented in Table 2, and the ROC curves of the proposed parameters are displayed in Figure 2.

**TABLE 2:** Diagnostic value of proposed parameters.

Parameters		Sensibility	Specificity	Positive predictive value	Negative predictive value	AUC
VEP Amplitude (μV)	OZ-60	0.999	0.625	0.829	0.999	0.909
	OZ-20	0.999	0.437	0.763	0.999	0.922
	OZ-12	0.999	0.294	0.707	0.999	0.897
OCT- GCC (μm)	Global GCC	0.521	0.999	0.999	0.614	0.858
	Bi-nasal of GCC	0.608	0.999	0.999	0.660	0.923
bi-nasal of GCC- VEP Amplitude 60′		0.619	0.999	0.999	0.652	0.807
bi-nasal of GCC- VEP Amplitude 20′		0.627	0.999	0.999	0.646	0.795
bi-nasal of GCC- VEP Amplitude 12′		0.600	0.999	0.999	0.630	0.905

VEP, visual evoked potential. OCT, optical coherence tomography. GCC, ganglion cell complex. Bi-nasal of GCC, bi-nasal sectors of GCC.

### Longitudinal study

All 61 eyes included from the 42 patient groups with PMA (23 female patients/19 male patients) were used in the follow-up study. Forty-four eyes were evaluated at 3 months and 30 eyes at 12 months post-surgical due to the non-attendance of the patients to one of the follow-up consultations (Table 3).

**TABLE 3:** Neuro-ophthalmological follow-up of patients with pituitary macroadenoma.

Evaluation	Parameter (unit)		Pre – surgical		Follow-up 3 months		*P*-value	Follow-up 12 months		*P*-value
			N (eyes)	Media ± sd	N (eyes)	Media ± sd		N (eyes)	Media ± sd	
	MD (dB)		55	9.6 ± 7	39	5.6 ± 6.1	1.2 × 10^–2^*	26	4.1 ± 5.6	4.4 × 10^–4^***
Psyco-physics	BCVA (VAR/**LogMAR**)		59	84 ± 18/**0.3**	39	90 ± 14/**0.2**	1.5 × 10^–1^	27	93 ± 10/**0.1**	2.3 × 10^–2^*
	CV		56	12 ± 8	39	12 ± 8	9.9 × 10^–1^	24	13 ± 9	5.1 × 10^–1^
	CS 1m		61	1.20 ± 0.30	39	1.20 ± 0.30	2.4 × 10^–1^	24	1.20 ± 0.3	2.7 × 10^–1^
	CS 3m		58	1.05 ± 0.30	39	1.20 ± 0.30	3.4 × 10^–1^	24	1.20 ± 0.3	3.4 × 10^–1^
VEP	Latency (ms)	OZ-60	35	119 ± 15	22	117 ± 9	6.6 × 10^–1^	25	112 ± 8	3.1 × 10^–2^*
		OZ-20	35	123 ± 14	22	127 ± 18	4.3 × 10^–1^	25	121 ± 14	6.3 × 10^–1^
		OZ-12	35	126 ± 14	21	129 ± 14	1.7 × 10^–1^	25	127 ± 15	2.1 × 10^–2^*
		O1-60	35	120 ± 17	22	122 ± 12	4.3 × 10^–1^	25	114 ± 10	1.7 × 10^–1^
		O1-20	35	123 ± 15	22	128 ± 19	2.6 × 10^–1^	25	122 ± 15	8.1 × 10^–1^
		O1-12	35	127 ± 16	21	130 ± 13	4.6 × 10^–1^	25	127 ± 16	8.5 × 10^–1^
		O2-60	35	118 ± 15	22	119 ± 13	8.6 × 10^–1^	25	114 ± 10	2.0 × 10^–1^
		O2-20	35	121 ± 14	22	129 ± 17	1.0 × 10^–1^	25	122 ± 15	9.5 × 10^–1^
		O2-12	35	122 ± 25	21	129 ± 14	2.7 × 10^–1^	25	127 ± 15	7.4 × 10^–1^
	Amplitude (μV)	OZ-60	35	4.9 ± 3.2	22	5.1 ± 2.2	3.9 × 10^–1^	25	7.5 ± 3.6	5.4 × 10^–3^**
		OZ-20	35	4.7 ± 3.8	22	4.9 ± 3.5	4.5 × 10^–1^	25	8.2 ± 4.6	1.8 × 10^–3^**
		OZ-12	35	4.2 ± 3.7	21	5.0 ± 4.0	1.2 × 10^–1^	25	7.3 ± 4.4	5.2 × 10^–3^**
		O1-60	35	3.6 ± 2.2	22	4.2 ± 2.0	1.4 × 10^–1^	25	5.1 ± 2.8	2.3 × 10^–2^*
		O1-20	35	3.2 ± 2.0	22	3.8 ± 2.4	2.2 × 10^–1^	25	5.1 ± 2.5	3.1 × 10^–3^**
		O1-12	35	4.0 ± 5.9	21	4.1 ± 2.4	9.4 × 10^–2^	25	4.9 ± 2.4	5.0 × 10^–3^**
		O2-60	35	3.6 ± 2.0	22	3.4 ± 1.9	7.8 × 10^–1^	25	5.0 ± 2.9	2.0 × 10^–2^*
		O2-20	35	3.0 ± 1.9	22	4.2 ± 2.9	1.1 × 10^–2^*	25	6.0 ± 3.0	9.4 × 10^–5^***
		O2-12	35	2.8 ± 2.0	21	3.8 ± 2.5	1.2 × 10^–1^	25	4.9 ± 2.8	4.2 × 10^–3^**
OCT	RNFL (μm)	RFNL	56	81 ± 14	44	75 ± 13	3.2 × 10^–2^*	30	71 ± 13	2.5 × 10^–3^**
		TS	56	50 ± 10	44	48 ± 9	3.5 × 10^–1^	30	44 ± 8	1.6 × 10^–3^**
		SS	56	102 ± 25	44	92 ± 26	5.5 × 10^–2^	30	87 ± 24	9.1 × 10^–3^**
		NS	56	63 ± 12	44	60 ± 8	1.9 × 10^–1^	30	59 ± 8	4.2 × 10^–2^*
		IS	56	107 ± 23	44	98 ± 25	7.1 × 10^–2^	30	97 ± 23	5.8 × 10^–2^
	GCC (μm)	Global GCC	56	67 ± 11	44	62 ± 10	2.5 × 10^–2^*	30	61 ± 12	1.3 × 10^–2^*
		RS1/LS3	56	71 ± 12	44	67 ± 12	8.4 × 10^–2^	30	65 ± 12	2.7 × 10^–2^*
		RS2/LS2	56	64 ± 12	44	60 ± 11	6.1 × 10^–2^	30	59 ± 13	2.8 × 10^–2^*
		RS3/LS1	56	62 ± 12	44	58 ± 11	2.5 × 10^–2^*	30	57 ± 14	2.8 × 10^–2^*
		RS4/LS6	56	60 ± 13	44	55 ± 11	2.0 × 10^–2^*	30	55 ± 13	1.4 × 10^–2^*
		RS5/LS5	56	67 ± 11	44	63 ± 11	3.6 × 10^–2^*	30	61 ± 13	8.0 × 10^–3^**
		RS6/LS4	56	75 ± 13	44	71 ± 12	1.0 × 10^–1^	30	69 ± 11	3.1 × 10^–2^*
		bi-nasal of GCC	56	61 ± 12	44	56 ± 11	2.1 × 10^–2^*	30	55 ± 17	1.0 × 10^–2^*

VEP, visual evoked potential. OCT, optical coherence tomography. MD, median deviation. BCVA, best-corrected visual acuity. CV, color vision. CS 1m, contrast sensitivity at 1m. CS 3m, contrast sensitivity at 3m. RNFL, retinal nerve fiber layer. GGCC, ganglion cell complex. Bi-nasal of GCC, bi-nasal sectors of GCC. O1. O2. Oz derivations. 60′-20′-12′ frequencies of stimulus. ***: *p* < 0.001, **: *p* < 0.01, *: *p* < 0.05. The maxima of analyzed eyes were 61, 44, and 30 for pre-surgical, 3-, and 12-month follow-up examinations.

#### Clinical examination

The main results from the neuro-ophthalmological follow-up evaluation of the PMA group are summarized in Table 3. MD was reduced significantly 3 months post-surgically (*p*_0–3 months_ < 0.05), and a year after, the mean value reaches half of the pre-surgical one (*p*_0–12 months_ < 0.01). BCVA improvement was appreciated a year after surgery (84 ± 18 vs. 93 ± 10 VAR, *p*_0–12 months_ < 0.05) while CV, CS 1m, and CS 3m remain statistically invariable.

#### Visual evoked potentials

Pre-surgical VEP P100 amplitude in OZ improved significantly for all spatial frequencies and derivations at 12 months post-surgery (0.001 < *p*_0–12 months_ < 0.05). Latency improved significantly only in one derivation (Oz) for 60′ and 12′ spatial frequencies at 12 months after surgery (*p* < 0.05) (Table 3).

#### Optical coherence tomography

The OCT evidenced that global GCC keeps declining postsurgery (baseline; 67 ± 11, 3 months; 62 ± 10, and 12 months: 61 ± 12; p _0–3 months_ < 0.05, p _0–12 months_ < 0.05), as well as bi-nasal of GCC (baseline; 61 ± 12, 3 months; 56 ± 11, 12 months; 55 ± 17; p _0–3 months_ < 0.05, p _0–12 months_ < 0.01) does too. RNFL showed a significant reduction at 3 and 12 months compared to pre-surgical evaluation (p _0–3 months_ < 0.05 and p _0–12 months_ < 0.01) (Table 3).

### Structure–function relation

The main parameters analyzed for the retinal structure included global/segmented GCC and RNFL. Visual function was assessed using visual field MD (subjective function) and VEP amplitudes (objective function). Table 4 shows the significant associations between analyzed parameters. There was a significant association between visual field MD and the VEP amplitude at 12 months after surgery in Oz at all spatial frequencies, being the strongest at 12′ (rho = 0.79, *p* < 0.001). Visual field MD showed also a significant association with global GCC and bi-nasal of GCC at 3 months (rho = 0.59 and rho = 0.57, *p* < 0.01). In addition, VEP amplitude Oz 12′ was significantly associated with global GCC and bi-nasal of GCC at 3 months (rho = 0.74 and rho = 0.71, *p* < 0.01). RNFL did not show any association with functional parameters (rho = 0.1, *p* = 0.152).

**TABLE 4:** Structure–function associations in pituitary macroadenoma.

Evaluation			rho	*p*-value
Visual field	VEP Amplitude Oz 3 months			
	MD 3 months	60′	–0.55	9.2 × 10^–3^**
		20′	–0.47	3.2 × 10^–2^*
		12′	–0.43	2.9 × 10^–3^**
	VEP Amplitude Oz 12 months			
	MD 12 months	60′	–0.62	4.8 × 10^–3^**
		20′	–0.66	2.0 × 10^–3^**
		12′	–0.79	5.4 × 10^–5^***
	Global GCC 3 months			
	MD 3 months	global GCC	–0.59	1.7 × 10^–3^**
		bi-nasal of GCC	–0.57	2.6 × 10^–3^**
VEP	Global GCC 3 months			
	Amplitude Oz 12′ 3 months	global GCC	–0.74	4.0 × 10^–3^**
		bi-nasal of GCC	–0.71	6.4 × 10^–3^**

VEP, visual evoked potential. MD, median deviation. GCC, ganglion cell complex. Bi-nasal of GCC, bi-nasal sectors of GCC. Oz derivation of VEP. 12′: frequency of stimulus. rho: Spearman’s coefficient. P-value: signification level. ***: *p* < 0.001, **: *p* < 0.01, *: *p* < 0.05.

**FIGURE 3:**
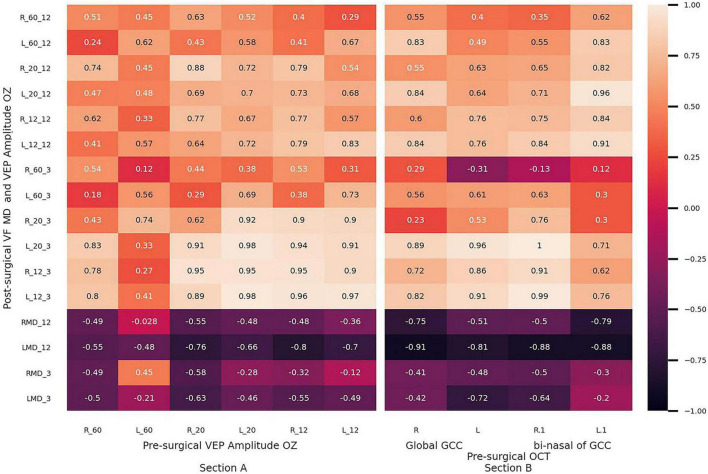
Thermic diagrams show rho values of Spearman’s correlations between pre-surgical and post-surgical parameters. **(Section A)** Pre-surgical visual evoked potential amplitude: amplitude OZ vs. post-surgical visual field median deviation and visual evoked potential amplitude OZ at 3 and 12 months. **(Section B)** Pre-surgical global and bi-nasal of ganglion cell complex vs. post-surgical visual field median deviation and visual evoked potential amplitude OZ at 3 and 12 months. L: left eye, R: right eye. Bi-nasal of GCC: bi-nasal sectors of a ganglion cell complex.

The predictive value of global GCC, bi-nasal of GCC, and the VEP amplitude in Oz of PMA patients’ vision recovery was estimated (Figure 3). Pre-surgical VEP amplitudes at 12′ and 20′ strongly correlated with the correspondent VEP amplitude values, determined at 3 months post-surgically (rho > 0.89), while it was moderate after a year (rho > 0.54) (Section A). Pre-surgical values of global GCC and bi-nasal of GCC exhibited moderate to strong correlations (rho > 0.6) with post-surgical values of 12′ VEP amplitude (Section B).

**FIGURE 4:**
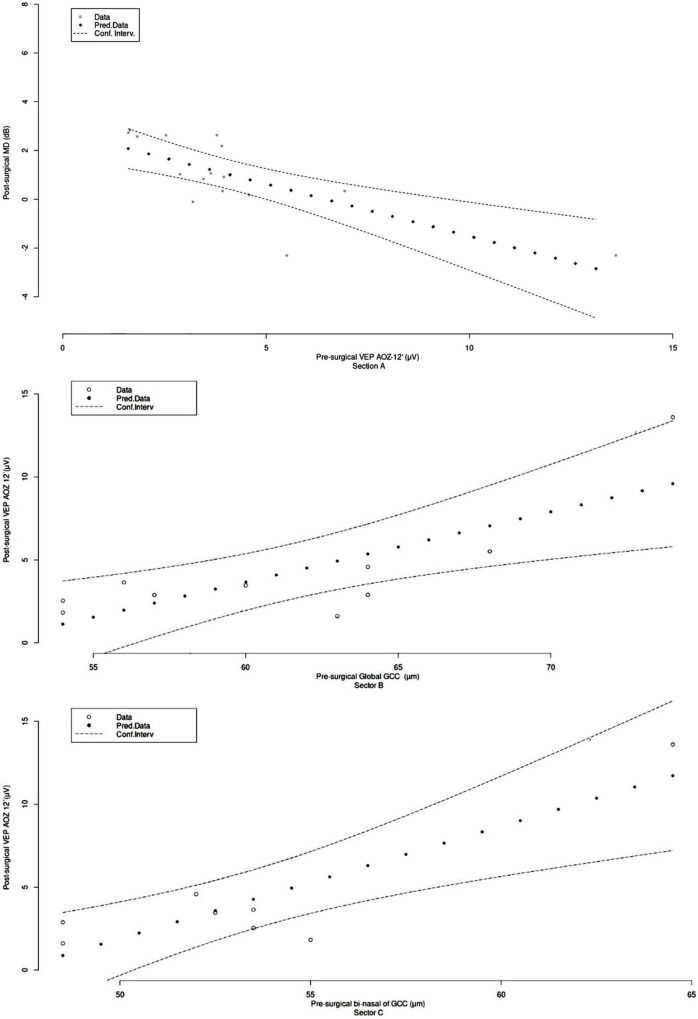
Predictive value of structure/function biomarkers in pituitary macroadenoma patients’ vision recovery. **(Section A)** Pre-surgical visual evoked potential amplitude Oz 12′ (X) vs. 3 months post-surgical visual field median deviation (Y), Y = 23 e^–0,8^ ^x^; *r*^2^ = 0.62. **(Section B)** Pre-surgical global ganglion cell complex (X) vs. 3 months post-surgical visual evoked potential amplitude OZ 12′ (Y), Y = 0.14 x – 8.4; *r*^2^ = 0.54. **(Section C)** Pre-surgical bi-nasal of ganglion cell complex (X) vs. post-surgical 3 months visual evoked potential amplitude OZ 12′ (Y), Y = 0.21 x – 9.1; *r*^2^ = 0.73.

Considering the abovementioned associations, prediction functions were obtained from best-fitted curves (Figure 4). VEP amplitude Oz at 12′ pre-surgical could determine visual field MD at 3 months by Y = 23 e^–0,8x^ with *r*^2^ = 0.62 (Sector A). Pre-surgical global GCC could predict VEP amplitude OZ 12′ at 3 months as Y = 0.14 x – 8.4 with *r*^2^ = 0.54 (Sector B). Pre-surgical bi-nasal of GCC could estimate the VEP amplitude OZ 12′ at 3 months by Y = 0.21 x – 9.1 with *r*^2^ = 0.73 (Sector C).

## Discussion

The present study demonstrated the importance of VEP P100 amplitude in association with the thickness of bi-nasal GCC sectors for the early diagnosis of visual pathway compression in PMA, as well as for monitoring and predicting visual recovery after surgery. Visual electrophysiology and optical coherence tomography are objective, sensitive, and non-invasive tools that provide relevant information on the visual pathway function in patients with PMA. The strengths of this investigation rely on the homogeneity and size of the sample, the high percentage of patients who attempted post-surgical evaluations, the inclusion of both eyes for the analysis since independence between eyes was demonstrated mathematically, and the integration of the data in individual as the unit of analysis by GEE.

### Cross-sectional study

Some authors have described that color vision loss is strongly associated with VF defects due to chiasm compression (Okamoto et al., 2010; Barzaghi et al., 2012; Yu et al., 2015; Sun et al., 2017). Slatkeviciene et al. (2016) reported that contrast sensitivity is approximately 2.4 times lower in PMA subjects than in healthy individuals (Slatkeviciene et al., 2016). In our study, BCVA, CV, CS 1m, and 3m were different between patients with PMA and healthy controls (*p* < 0.001). Five patients did not show visual field defects in the pre-surgical examination, which is consistent with what has been described by other authors (Yum et al., 2016; Tieger et al., 2017; Blanch et al., 2018). In the rest of our PMA patients with visual field defects (88%), MD was a reliable parameter to detect compressive damage (Khaliliyeh et al., 2016; Beltrame et al., 2018).

Neuro-ophthalmologists address the suspicion of PMA by signs and symptoms by the evaluation of several parameters using structural and functional tests. MRI identifies the presence of an abnormal mass in the sellar region, but it neither allows for quantifiable measurement of nerve fiber damage nor visual function. The diagnosis of PMA is confirmed by the pathological evaluation of surgically removed tumors. Among functional tests, VEP has been extensively used regarding latency, which reflects conduction velocity along the demyelinated segment of nerve fibers, which is the biomarker of choice. Previous studies have considered the VEP P100 latency as a diagnostic marker of early functional changes in the visual pathway before the appearance of other clinical features in PMA (Popescu et al., 2021). VEP represents an extension of clinical examination but is not routinely performed in clinical settings. In that sense, the amplitude of VEP has been scarcely explored (Lachowicz and Lubiński, 2018). In the present study, VEP P100 amplitude discriminated between patients with PMA and controls. GEE analysis evidenced a probability approximately 2.5-fold of being classified as a patient with PMA when individuals show decreased VEP amplitude and typical clinical signs and symptoms. Bootstrap analysis revealed that the VEP P100 peak amplitude was a more reliable parameter than latency at 60′ and 12′ (AUC _Amplitude 60′_ of 0.909 vs. AUC _Latency 60′_ 0.860, *p* = 0.049 and AUC _Amplitude 12′_ 0.897 vs. AUC _Latency 12′_ 0.648 *p* = 0.021), for PMA diagnosis.

The use of OCT-measured peripapillary RNFL and macular thickness segmentation to quantify axonal loss has been previously documented in chiasm compressive lesions, as well as associations between RNFL/GCC thinning and visual field (Monteiro et al., 2014; Danesh-Meyer et al., 2015; Tieger et al., 2017; Meyer et al., 2022). In addition, Yum et al. (2016) evidenced the diagnostic ability of global GCC for detecting early structural retinal changes following PMA chiasm compression, in which they selected the worse eye as the unit of analysis and detected that the superonasal sector of GCC showed a statistically significant detection rate in the PMA group. In such investigations, the AUC related to the inferonasal and superonasal sectors of GCC thicknesses was better than global GCC thicknesses for discriminating between the preperimetric PMA and controls (Yum et al., 2016). In the present study, the bootstrap analysis confirmed the findings of Agarwal et al. and Tieger et al., because global GCC was a more reliable parameter than global RFNL (AUC _GCC_ 0.858 vs. AUC _RNFL_ 0.781, *p* = 0.018) (Tieger et al., 2017; Agarwal et al., 2021). In addition, the bi-nasal of GCC was the most useful biomarker for PMA diagnosis (AUC_bi–nasal of GCC_ 0.923 vs. AUC _ST of RNFL_ 0.798, *p* = 0.045 and AUC_bi–nasal of GCC_ 0.923 vs. AUC _GCC_ 0.858, *p* = 0.023). GEE revealed a 2-fold probability of being a patient with PMA when signs and symptoms are present and global GCC thickness is reduced, particularly in the bi-nasal GCC areas.

### Longitudinal study

Visual recovery after transsphenoidal surgery occurs in various phases. Initial improvement may be from minutes to a few days. Additional significant changes may continue for months. There is no consensus among clinicians regarding how frequently patients with PMA should be followed post-surgery. Some authors suggest MRI follow-up every 1–2 years (Lithgow et al., 2019). PMA follow-up studies reviewed in the literature have several limitations: (i) The number of subjects that attempt the consultation is quite reduced, (ii) there is no consensus regarding the moment of evaluation, and (iii) there is significant variability in test and biomarkers selection (Tieger et al., 2017; Taghvaei et al., 2019; Wang et al., 2020; Agarwal et al., 2021). In our study, there were significant differences in BCVA between pre-surgical and 12-month examinations (*p* < 0.05), and similar results were reported by Taghvaei et al., at 3-month evaluation; conversely, Wang et al. found no differences after a 2-year follow-up (Taghvaei et al., 2019; Wang et al., 2020). Visual field MD has been evaluated to estimate visual recovery by almost every author. Wang et al. (2020) evaluated patients with several types of lesions compressing the optical chiasm and found differences as early as 6 weeks after surgery but no differences with subsequent evaluations. Our pre-surgical values of color vision and contrast sensitivity were similar to the report by Wang et al. Post-surgical recovery does not improve between 3- and 12-month follow-up.

Taghvaei et al. (2019) classified VEP as a helpful quantitative and objective ancillary test for assessing postoperative visual improvements in patients with PMA, but they did not find a significant increase in amplitude 3 months after surgery. The authors recognized as limitations of such a study: small sample size and lack of structural visual pathway assessment. Our research evidenced a significant recovery of magnocellular and parvocellular traffic 12 months after surgery and the usefulness of VEP amplitude at every derivation (O1, O2, and Oz) and stimulus frequency (60′, 20′, and 12′), as a biomarker for assessing it.

Danesh-Meyer et al. described improved visual outcomes in patients with pre-surgical RNFL thickness above 80 μm (Danesh-Meyer et al., 2015). Tieger et al. (2017) and Blanch et al. (2018) evaluated the global GCC as a possible marker for surgical decompression. Tieger et al. described persistent damage in the nasal sector at 1-year follow-up in eight patients that attended surveillance visits (Tieger et al., 2017). Other authors also reported the thinning of RFNL and global GCC (Moon et al., 2011; Salah and Ali, 2018; Poczos et al., 2022). Our results presented in Table 3 data are consistent with the abovementioned findings, and global RNFL and, in less degree, GCC still decrease after surgery (p < 0.05). It could be interpreted as visual function improves despite the lack of recovery of GCC thickness because compressive lesion blocks the transmission of impulses in the optic nerve axons that have not been destroyed yet. Then, after decompression, nerve transmission can be restored in those axons that survived the sustained injury but still a subset of ganglion cells will go into apoptosis (Horton, 2017; Pietrucha-Dutczak et al., 2018).

Several authors have suggested that an RNFL thinning could be an indicator of optic nerve atrophy secondary to compression, but it does not imply that ganglion cells were lost (Moon et al., 2011; Danesh-Meyer et al., 2015; Salah and Ali, 2018). Retinal ganglion cell death occurs after axonal injury and seems to depend on the initiation of apoptosis due to the failure of neuroprotective mechanisms (Levin, 1999; Horton, 2017). Retinal cells are closely interacting with each other and the death of a single cell releases glutamate which triggers an excitotoxic cascade, resulting in nitric oxide production and other metabolite deregulation affecting the survival of surrounding cells (Pietrucha-Dutczak et al., 2018). In the present study, global GCC thickness decreased over time; such reduction was less significant than RFNL thinning, which is in accordance with Salah’s study (Salah and Ali, 2018). VEP amplitude increasing after decompression due to axonal traffic unblocking was more significant than structural variation, so electrophysiology sensed vision recovery better than OCT.

### Structure–function relationship and visual recovery prediction

Few groups have explored associations between pre-surgical structural/functional markers in patients with PMA. Multifocal VEP parameters and macular volume by OCT have been compared by Sousa et al. Their findings indicated a moderate correlation between amplitudes with macular volume and peripapillary RNFL thickness. This study had the limitation of the relatively small number of patients included (27 eyes/21 patients) and that multifocal VEP is a pretty laborious technique (Sousa et al., 2017). Tieger et al. reported associations between RNFL and GCC with MD, but they were very discrete (*r*^2^ = 0.15 and 0.25) (Tieger et al., 2017). Taghvaei et al. (2019) reported that VEP P100 wave latency and amplitude significantly correlated with static automatic perimetry score and BCVA. In our study, structural parameters were associated with functional ones 3 months after surgery. In addition, significant correlations between VEP amplitude in OZ and MD were obtained in the 12-month evaluation.

Predicting which patients are likely to have favorable visual outcomes is important as this could help decision-making regarding surgical intervention. Multiple reports using multivariate analyses have demonstrated that age and tumor size are not significant visual predictors (Müslüman et al., 2011; Lee et al., 2013; Hisanaga et al., 2017). Neither Agarwal et al. nor Tieger et al. were able to statistically analyze the predictive capacity of OCT parameters in visual recovery estimation due to the limited number of patients (five and eight eyes, respectively) attending follow-up (Tieger et al., 2017; Agarwal et al., 2021).

Poczos et al. (2022) evaluated the predictive value of pre-surgical VEP amplitude and GCC thickness for estimating 6 months post-surgical VF MD after decompression in a heterogeneous sample (16 patients); GCC exhibited a moderate association with VF MD (rho = 0.63, *p* < 0.01), but no association was found with VEP amplitude (rho = 0.26, *p* > 0.05). There is no consensus in the literature about what to measure and when it should be done. In the present study, as stated, OCT and perimetry should be done before surgery and usually at 3 months. A few authors have used VEP for monitoring post-surgical visual recovery without success because follow-up periods were shorter than in the present study. VEP amplitudes should be evaluated previous to surgery for predicting short-term recovery (3 months), but further, significant, objective visual function recovery is observed after 12 months.

Henceforth, equations for the estimation restoration of visual pathways have not been reported. Thus, considering the association evidenced in thermic maps (Figure 3), best-adjusted curves for subjective and objective function prediction were calculated (Figure 4) with 0.54 < r^2^ < 0.74. These results suggest that pre-surgical bi-nasal of GCC and VEP amplitude could predict visual recovery in the short term. Prediction of post-surgical visual outcomes allows the physician to perform personalized counseling. Therefore, patients will be able to plan regarding work, family, and social routines.

## Final remarks

Bi-nasal sectors of the ganglion cell complex measured by OCT and VEP P100 amplitude are efficient biomarkers of axonal loss in PMA patients’ diagnosis. VEP P100 amplitude is suitable for monitoring patients’ evolution. Pre-surgical values of global GCC, bi-nasal GCC, and VEP P100 amplitude in Oz are strongly associated with post-surgical values of VEP amplitude and visual field MD. Pre-surgical data related to these biomarkers could help predict the restoration of parvocellular pathway traffic after decompression. Some equations have been obtained to estimate visual recovery whose validation is in progress and could improve personalized decision-making protocols.

## Data availability statement

The raw data supporting the conclusions of this article will be made available by the authors, without undue reservation.

## Ethics statement

The studies involving human participants were reviewed and approved by Ethics Committee of Cuban Eye Institute “Ramon Pando Ferrer.” The patients/participants provided their written informed consent to participate in this study.

## Author contributions

OH-E and EC-L were conceived, designed, and conducted the study. MP-B and HG-D performed all the statistical analyses. OH-E and JG-G were collected the data. CM-S and HG-D reviewed the design and data interpretation. OH-E wrote the first draft of the manuscript. EC-L edited the final version of the manuscript. All authors contributed to the manuscript revision, read, and approved the submitted version.
